# Supplementary material for: Remote evaluation of STH program coverage: Experiences from the DeWorm3 study, India
Source: PLoS Negl Trop Dis. 2023 Nov 16;17(11):e0011748. doi: 10.1371/journal.pntd.0011748 (PMC10653432; doi:10.1371/journal.pntd.0011748)
Supplement: S1 File — (DOCX) [file pntd.0011748.s001.docx]

**Supporting File 1: Coverage Evaluation Survey Questionnaire and Qualitative Guideline**

**Annex 1: Coverage Evaluation Survey – Questionnaire**

| **S. No** | **Questions** |
| --- | --- |
| 1. | Did someone give you a tablet like this in the past month to treat worms (at home, at school or in the community)? |
| 2. | If, refused, why didn't you receive the tablet? |
| 3. | Did you swallow the tablet? |
| 4. | Why did you not swallow the tablet? |
| 5. | Why did you swallow the tablet? |
| 6. | Did you swallow the tablet in front of the person who gave it to you? |
| 7. | Who gave you the tablet? |
| 8. | Where did they give you the tablet? |
| 9. | Did you feel any side effects after swallowing the tablet? |
| 10. | Which of the following statements are true about side-effects you experienced after swallowing the tablet? |
| 11. | How many of these tablets did you swallow? |
| 12. | Were you aware that tablets would be distributed before it happened? |
| 13. | How did you know the distribution was going to happen? |
| 14. | Did any of your neighbours take the distributed tablet? |
| 15. | What did you like about the community treatment programme? |
| 16. | What did you not like about the community treatment programme? |
| 17. | Next time the tablets are given, how would you want them distributed? |
| 18. | Did you have to change your daily routine to participate in the treatment day? |
| 19. | About how much of your time did it take to participate in the treatment day, including the time you spent waiting for the drug distributor to arrive? |
| 20. | About how many minutes did the drug distributor spend at your house on the treatment day? |

**Annex 2: Coverage evaluation survey: FGD question guide**

| **S.No** | **Question** |
| --- | --- |
| 1. | Can you describe the process of conducting face to face survey, the steps involved in carrying out face to face survey? How did you plan for face-to-face survey, please describe in detail? |
| 2. | Can you describe the process of conducting phone survey, the steps involved in carrying out phone survey? How did you plan for phone-survey, please describe in detail? |
| 3. | What are the advantages and disadvantages of conducting coverage survey using mobile phones compared to in-person interviews? *Probe (if needed):* Establishing contact, time taken to locate houses; time taken to complete phone/face-to-face survey, achieving survey coverage |
| 4. | What are the advantages/disadvantages in reaching members within a household in phone survey compared to face-to-face survey? *Probe (if needed):* Proxy responses, gender of respondents, reach to all household members |
| 5. | Please share you experience of consenting HH for the interview in phone and face-to-face surveys? *Probe (if needed):* What was easy and why? |
| 6. | Please share your experience of asking the coverage survey questions and if there was any difference when the questions were asked over phone compared to face-to-face surveys? *Probe (if needed):* Which method has better data quality and why? Were there any specific questions which were difficult to comprehend by HH or they took time to respond in face-to-face/phone survey? |
| 7. | In your opinion, how feasible is it to conduct a phone survey in the future even if there is no lock down? Which method would you prefer between face-to-face/phone survey for conducting cMDA coverage surveys in future and reasons why? |
