# Supplementary material for: Remote evaluation of STH program coverage: Experiences from the DeWorm3 study, India
Source: PLoS Negl Trop Dis. 2023 Nov 16;17(11):e0011748. doi: 10.1371/journal.pntd.0011748 (PMC10653432; doi:10.1371/journal.pntd.0011748)
Supplement: Attachment — Submitted filename: Reviewer_comments.docx [file pntd.0011748.s003.docx]

**Reviewer Comments**

This study describes the experiences of implementing a coverage evaluation surveys (CES) via mobile phones, including challenges and best practices compared to standard in-person CES implementation. Authors suggested some advices for using mobile phone-based CES as alternative tools to validate the treatment coverage of community mass drug administration when access to households was restricted. To improve the quality of responses and optimize phone-based CES authors suggested to confirming phone numbers while recording them, to collecting additional household phone numbers including those of women, and avoiding open-ended questions during the census step. This is certainly and interesting alternative coverage survey method and will need to be published. However, the following major concerns have emerged after review of the manuscript and will need to give more details/precisions:

This study was to be approached in two phases: Quantitative and Qualitative.

Quantitatively, we were hoping to see benchmarking of standard in-person CES previous used and phone-based CES, which will allow us to analyze the effectiveness of several parameters targeted in this study. What is the number of households called per cluster? for how many households was the call successful/contacting? Consenting? how many of repondents? … In this case a flow diagram of the selection of households would be necessary.

At the qualitative level: it is mentioned in Study design and data collection that "eight CES implementers, six from Timiri and two from Jawadhu hills sub-site, had experience of implementing both in-person and phone-based CES. All were invited to participate in a focus group discussion (FGD) conducted at each of the two sub-sites with two social scientists facilitated the interviews using a semi-structured question guide (S1file-Annex 2)". We understand that only two focus groups were carried out (1FGD in Timiri and 1FGD in Jawadhu). We understand also that the two CES implementers of Jawadhu participated in the FGD of Timiri and that the 6 CES implementers of Timiri also participated in the FGD of Jawadhu with the same questionnaire. Why didn't you think about creating the FGD of each sub-site with the CES implementers of each sub-site? Please clarify.
