# Supplementary material for: Remote evaluation of STH program coverage: Experiences from the DeWorm3 study, India
Source: PLoS Negl Trop Dis. 2023 Nov 16;17(11):e0011748. doi: 10.1371/journal.pntd.0011748 (PMC10653432; doi:10.1371/journal.pntd.0011748)
Supplement: Attachment — Submitted filename: Response to Editors comments_final.docx [file pntd.0011748.s004.docx]

**Response to Editor’s comments**

Thank you very much for your feedback. Responses to the comments are given below in bold.

1) Please ensure that the full contributions of each author are acknowledged in the "Add/Edit/Remove Authors"" section of our submission form.

**Thank you. We have updated the CRediT author contributions.**

2) We have noticed that you have uploaded "S2 file .doc" file as supporting information but you have not included it in the list of legends.  Please add a citation of this SI data file into the list of legends of your supporting information files.

**Thank you very much. We have included the S2 file in the list and added the citation for S1 and S2 files.**

3). Please note that if child participants, the statement must declare that formal consent (must state whether verbal/written) was obtained from the parent/guardian.

**There were no child participants in the focus group discussions of this study.**

4) Potential Copyright Issues:
1) [Figure1,2]: Please confirm whether you drew the images / clip-art within the figure panels by hand. If you did not draw the images, please provide (a) a link to the source of the images or icons and their license / terms of use; or (b) written permission from the copyright holder to publish the images or icons under our CC BY 4.0 license.

**Thank you for your feedback. We have revised the images in figures 1 & 2 from the following links –**

1. [https://openclipart.org](https://openclipart.org/)

<https://openclipart.org/detail/115159/ftdopewarspill>

<https://openclipart.org/detail/254036/human-family>

2. [https://www.freepik.com/icon/student_185568#fromView=resource_detail&position=11](https://www.freepik.com/icon/student_185568)
3. <https://icon-library.com/icon/person-on-phone-icon-27.html>

2) [Figure 3]: please (a) provide a direct link to the base layer of the map (i.e., the country or region border shape) and ensure this is also included in the figure legend; and (b) provide a link to the terms of use / license information for the base layer image or shapefile.

**We did not use any external base map. All features in the map were recorded by trained Deworm3 field workers in both study sites. The map was made using ArcGIS 10.3.1 for Desktop, a Geographic Information System (GIS) software developed by ESRI. ArcGIS 10.3.1 for Desktop. (2015). Esri.** [**https://www.esri.com**](https://www.esri.com)
