# Supplementary material for: Remote evaluation of STH program coverage: Experiences from the DeWorm3 study, India
Source: PLoS Negl Trop Dis. 2023 Nov 16;17(11):e0011748. doi: 10.1371/journal.pntd.0011748 (PMC10653432; doi:10.1371/journal.pntd.0011748)
Supplement: Attachment — Submitted filename: Editors & Reviewers Comments.docx [file pntd.0011748.s005.docx]

October 16, 2023

Uwem Friday Ekpo and Francesca Tamarozzi

Editors

PLOS Neglected Tropical Diseases (NTDs)

Re: PLOS Neglected Tropical Diseases submission (PNTD-D-23-00543) - [EMID:e82764504100377c]

Dear PLOS NTDs Editors,

We sincerely thank you and the reviewers for their comments on our manuscript titled: "Evaluating STH program coverage remotely - Experiences from the DeWorm3 study in India" Responses to the comments are given below. We have revised the manuscript in response to the comments and feedback and submitted both tracked and clean version.

Sincerely,

Sitara Swarna Rao Ajjampur

The Wellcome Trust Research Laboratory, Division of Gastrointestinal Sciences, Christian Medical College, Vellore, India

Phone: +91-416-228-2052

Fax: +91-416-228-2486

Email: [sitararao@cmcvellore.ac.in](mailto:sitararao@cmcvellore.ac.in)
Thank you very much for your feedback. Responses to the comments are given below in bold.

Editors Comments
Adding quantitative analyses to this manuscript and to apprise critically the results in the discussion would improve the information gathered on this topic and be of benefit to readers of PLoS NTD

**Thank you for the valuable suggestion that has helped improve our paper.**

**We have included household sampling and participation quantitative data as a new figure (Figure 3) and described these data and analysis in the relevant sections. This showed that households reached from sample lists in phone-based CES was much lower (56%) compared to in-person CES (89%) because phone numbers were wrongly recorded, or calls were unanswered. Therefore, a higher number of households (3,600) had to be sampled in order to reach the sample size of 2,000 households in phone-based CES compared to 2,352 households in in-person CES. Observations on gender bias and proxy responses in the phone based CES were also supplemented with quantitative analysis. Women’s participation in CES was significantly lower in phone-based CES (66%) compared to in-person (94%) CES (Z = -22.38; p<0.01). In confirming treatment compliance from family members, significantly higher proportion of households gave proxy response in phone-based CES (51%) than in-person CES (21%) (Z = 20.23; p<0.01).**

**Reviewer comments**

**Methods**
Reviewer #1: (No Response)
Reviewer #2: This study suggest the phone-based overage evaluation survey CES as a viable option to evaluate treatment coverage when in-person coverage evaluation surveys was issue when access to households was restricted as it is the case during the COVID-19 pandemic. The objectives of the study are clearly articulated with a clear testable hypothesis stated and the study design is appropriated to address the stated objectives. Globally the method is fine but should be improved.
**Thank you very much.**

**Results**
Reviewer #1: (No Response)
Reviewer #2: Interestingly, the results are clearly and completely presented.
**Thank you very much.**

**Conclusions**
Reviewer #1: (No Response)
Reviewer #2: Yes, the conclusions suggested are supported by the data presented and authors discuss how the data can be helpful to improve the new method suggested quality. Authors point out a number of challenges with phone-based CES methods: high non-response rates, gender bias in response and, the challenge to obtaining responses to open-ended questions.
**Thank you very much.**

**Summary and General Comments**
Reviewer #1: The author did not make any substantial change to the paper: The paper in the present version address only the opinions of the users without any information on performance and cost of the tools. Since the authors are mentioning that the tool performances are addressed in a different paper, I suggest the authors to join the two papers in a single one with all the relevant information. This will allow the reader to judge about the applicability of the tool.

**Thank you for the suggestion.**

**We have included household sampling and participation quantitative data as a new figure (Figure 3) and described these data and analysis in the relevant sections. This showed that households reached from sample lists in phone-based CES was much lower (56%) compared to in-person CES (89%) because phone numbers were wrongly recorded, or calls were unanswered. Therefore, a higher number of households (3,600) had to be sampled in order to reach the sample size of 2,000 households in phone-based CES compared to 2,352 households in in-person CES. Observations on gender bias and proxy responses in the phone based CES were also supplemented with quantitative analysis. Women’s participation in CES was significantly lower in phone-based CES (66%) compared to in-person (94%) CES (Z = -22.38; p<0.01). In confirming treatment compliance from family members, significantly higher proportion of households gave proxy response in phone-based CES (51%) than in-person CES (21%) (Z = 20.23; p<0.01).**

Reviewer #2: Globally the reviewers suggestions have been duly incorporated and response have been given accordingly. Therefore, this manuscript could be accepted for publication in Plos NTD
**Thank you very much.**
